# Patients’ sense of responsibility to healthcare providers and its predictors: A national cross-sectional survey in China

**DOI:** 10.1371/journal.pone.0207361

**Published:** 2018-12-05

**Authors:** Beizhu Ye, Xinzi Wang, Fang Wang, Ping Zhang, Yao Cheng, Yi Sun, Hongwei Jiang, Hua Qin, Aiguo Liu, Yang Liu, Xi Zhu, Naixing Zhang, Yuan Liang

**Affiliations:** 1 Department of Social Medicine and Health Management, School of Public Health, Tongji Medical College, Huazhong University of Science and Technology, Wuhan, Hubei, China; 2 International Baccalaureate Diploma Program; Wuhan British-China School, Wuhan, Hubei, China; 3 Department of Epidemiology and Biostatistics, School of Public Health, Tongji Medical College, Huazhong University of Science and Technology, Wuhan, Hubei, China; 4 Department of Gastroenterology, Tongji Hospital, Tongji Medical College, Huazhong University of Science and Technology, Wuhan, Hubei, China; 5 Department of Pediatrics, Tongji Hospital, Tongji Medical College, Huazhong University of Science and Technology, Wuhan, Hubei, China; 6 Department of Pancreatic Surgery, Union Hospital, Tongji Medical College, Huazhong University of Science and Technology, Wuhan, Hubei, China; 7 Department of Health Management and Policy, College of Public Health, the University of Iowa, Iowa City, Iowa, United States of America; 8 Department of Medical Administration, Health and Family Planning Commission of Shenzhen, Shenzhen, Guangdong, China; Medical University Graz, AUSTRIA

## Abstract

**Objectives:**

To evaluate patients’ sense of responsibility to healthcare providers and to determine its predictors using on a national sample in China.

**Methods:**

We conducted a national cross-sectional survey in China with a stratified cluster sample of patients treated in 77 hospitals between July 2014 and April 2015. Patients’ sense of responsibility to healthcare providers was measured with four questions assessing patients’ perceptions regarding their responsibilities to respect doctors, respect nurses, coordinate with health professionals, and comply with hospital rules. Predictors included patient sociodemographic characteristics and their past hospitalization experience.

**Results:**

Small proportions of respondents reported that they perceived having no responsibility to respect doctors (8.9%), respect nurses (7.9%), comply with hospital rules (6.7%), or coordinate with health professionals (6.3%). Multivariate regression analyses showed that the strongest predictor of patients’ sense of responsibility to healthcare providers was patinets’ trust in health professionals, followed by patients’ education level. Familiarity with healthcare professionals and past hospitalization frequency were inversely associated with patients’ sense of responsibility to healthcare providers.

**Conclusions:**

Although only a small proportion of the patients reported feeling no or low sense of responsibility to healthcare providers, the lack of respect and collaboration from these patients can negatively affect patient-provider relationships. Healthcare administrators need to communicate clearly with the patients and the public about the role of patients and the limitations of medicine in order to instill a sense of patients’ responsibility.

## Introduction

In the past several decades, fundamental social changes in China have resulted in increasing empowerment of individuals, creating a surge in rights-based movements, especially for the rights of consumers [1–3]. However, responsibility, the counterpart of rights, has not received the same attention as individuals adapted to the new consumerism culture. In the medical field, this phenomenon is reflected by the fact that patients are concerned about their rights as consumers without a balancing sense of responsibility to healthcare providers [4], which has resulted in the deterioration of patient–provider relationships. Additionally, individuals increasingly use Internet resources to access health- and healthcare-related information, which often consists of false or misleading information related to patient-provider relationships, even with the intense Internet regulation and censorship in China [5–7]. Without a sense of patients’ responsibility, the information environment might further impair the mutual understanding between patients and providers.

Previous studies on patient-provider relationships have placed an emphasis on the responsibility of healthcare providers to patients in the medical treatment process [8–12], including improvement of professional skills and quality of healthcare services, and informing and involving patients in their medical decisions. However, as the recipients of health services, patients also have a responsibility to the healthcare providers. In recent years, some researchers have become interested in patients’ responsibility, but the emphasis has been on the responsibility of patients for their own health [13–15]. Some scholars have argued that patients should have responsibilities that include expressing respect and gratitude to the providers of health care [16–19]; however, empirical data are limited regarding patients’ sense of their responsibility to healthcare providers.

To understand patients’ sense of responsibility to health service providers and to determine its predictors, we conducted a national survey among inpatients of general hospitals in China. Notably, in this period of social transition from a planned to a market economy [20], health affairs in China are complex [21–22]; China’s experience, including those regarding the protection of patients’ and healthcare providers’ rights, may thus provide an important reference for both developing and developed countries.

## Methods

The study was approved by the Medical Ethics Committee of the School of Public Health, Tongji Medical College, Huazhong University of Science and Technology.

This study is based on a stratified cluster sampling survey conducted at secondary and tertiary hospitals across the mainland China. The details of this survey have been described in a previous report [23]. Briefly, we selected six provinces (Gansu, Yunnan, Jiangsu, Shandong, Hubei, and Guangdong) and metropolitan Beijing, China’s capital, which have a combined population of 427.15 million, accounting for 31.88% of the total population of China [24]. According to National Bureau of Statistics of China, per capita GDP of Beijing, Guangdong, Jiangsu and Shandong was close to or above $10,000 in 2014, which was higher than the national average. There was a total of 85 eligible hospitals in the selected regions, of which 8 refused to participate, leaving a total of 77 participating hospitals (90.59%). In each hospital, convenience sampling was used to select patients from three to four surgical departments of different specialties and another three to four internal medicine departments (excluding obstetrics and pediatrics). A total 528 departments were involved and all inpatients in these departments during the study period were surveyed. Data were collected from July 2014 to April 2015. The survey was a self-administered written survey. All participants provided verbal informed consent. There were 24,250 eligible participants, of whom 11,884 did not complete the survey (49.01%). We excluded 4,128 (17.02%) completed questionnaires that contained apparent fraudulent or erratic responses (e.g., two questionnaires filled in with identify handwriting, a questionnaire containing conflicting responses to different questions) after three trained research assistants conducted a manual check for handwriting and a computer-assisted quality assurance check during data entry. Our analysis used data from the 8,238 remaining responses (response rate = 33.97%). There was no systematic difference between our sample and population in the seven selected regions on demographic characteristics except the education level: the sample has a slightly higher average education level than that of the population potentially due to response bias.

### Measures

With reference to previous studies [16,25], we determined patients’ sense of responsibility to healthcare providers using four questions: (1) “In general, do you think patients have a responsibility to respect doctors?” (Responsibility to respect doctors); (2) “In general, do you think patients have a responsibility to respect nurses?” (Responsibility to respect nurses); (3) “Do you think patients have a responsibility to coordinate with health professionals in the medical treatment process?” (Responsibility to coordinate with health professionals); (4) “Do you think patients have a responsibility to comply with hospital rules?” (Responsibility to comply with hospital rules). These questions demonstrated high internal consistency (Cronbach α = 0.935). Participants were asked to indicate their response to each question using a 5-point scale: I don’t know, Not at all, Very little, A little, A fair amount/A lot.

Familiarity with health professionals was measured using the question, “How many of your relatives are health professionals?” (coding: 0 = No, 1/2/3/≥4 = Yes). Hospitalization within the past 3 years was determined using the question, “How many times have you been hospitalized in the last 3 years, not including this time?” (coding: 0 = No, 1/2/3/≥4 = Yes). Trust in health professionals was measured with the question, “How many health professionals are trustworthy, in your opinion?” (Trust variable coding: Very few/A few/Generally/quite a few = Low; Many/Most = High).

Sociodemographic characteristics included sex (male, female), age (≤30, 30–44, 45–59, ≥60 years old), education level (middle school and below, high school, bachelor’s degree and above), marital status (married, unmarried or other), medical insurance (yes, no), and financial status (good, fair, poor).

### Statistical analyses

The chi-square test was used to assess differences in patients’ sense of responsibility according to sociodemographic characteristics (including sex, age, education level, marital status, medical insurance, and financial status), familiarity with health professionals, hospitalization within the past 3 years, and trust in health professionals. Bivariate logistic regression models were used to estimate the association between predictors (social demographic characteristics and past hospitalization experience) and four dichotomous outcome variables. We used OR and 95%CI to indicate the likelihood of much responsibility. All analyses were performed using SPSS, version 22.0 (SPSS Inc., Chicago, IL, USA).

## Results

Table 1 shows the distribution of patients’ sense of responsibility across the variables of demographic characteristics. In general, the most frequent response to the four questions regarding patients’ sense of responsibility to healthcare providers was "Fair/A lot" (79.9%–83.1%); however, responses of “I don’t know” (unaware of any responsibility) and “Not at all” (feeling no responsibility) are noteworthy. The proportion of patients with no responsibility (combined responses of “I don’t know” and “Not at all”) to respect doctors was 8.9%; to respect nurses was 7.9%, to comply with hospital rules was 6.7%, and to coordinate with health professionals was 6.3%, in descending order. For combined responses of “A little” and “Very little” responsibility to healthcare providers, the proportion for patients’ sense of responsibility to comply with hospital rules was 12.4%, to respect doctors was 11.8%, to respect nurses was 11.8%, and to coordinate with health professionals was 10.8%, in descending order. Overall, one-fifth of patients perceived little or no responsibility, with the lowest proportion for sense of responsibility to respect the doctor (20.6%) and the highest for sense of responsibility to coordinate with health professionals (17.1%). As shown in Table 1, the distribution of the 4 outcomes across education level, medical insurance and financial status were significantly different.

**Table 1:** Social demographic characteristics and patients’ sense of responsibility to healthcare providers.

Social demographic characteristics	n(%)	Responsibility to respect doctors (n = 7867)						Responsibility to respect nurses (n = 7928)						Responsibility to coordinate with medical professionals (n = 7901)						Responsibility to comply with hospital rules (n = 7860)					
		Don’t know %	Not at all %	Very little %	A little %	Fair/ much %	*p*	Don’t know %	Not at all %	Very little %	A little %	Fair/ much %	*p*	Don’t know %	Not at all %	Very little %	A little %	Fair/ much %	*p*	Don’t know %	Not at all %	Very little %	A little %	Fair/ much %	*p*
**Total**	8238(100.0)	4.9	4.0	5.4	6.4	79.4		4.3	3.6	5.4	6.4	80.3		3.1	3.2	4.6	6.2	82.9		3.4	3.3	5.2	7.2	81.0	
**Gender**																									
** Female**	3602(52.6)	4.7	3.7	5.4	6.3	79.9	0.973	4.4	3.4	5.1	6.4	80.8	0.676	2.8	3.1	4.5	6.3	83.2	0.826	3.2	3.3	5.0	7.2	81.3	0.856
** Male**	3250(47.4)	4.1	3.9	5.1	6.4	79.9		3.8	3.5	5.5	6.4	80.7		3.1	2.9	4.4	5.9	83.7		3.2	3.0	5.2	6.8	81.8	
**Age(years)**																									
** ≤30**	1040(14.6)	6.1	3.6	4.9	6.4	79.0	0.095	4.8	3.8	5.3	5.9	80.2	0.263	3.3	3.4	4.7	5.3	83.3	0.096	3.7	3.7	4.1	6.8	81.8	0.236
** 30–44**	1517(21.3)	5.5	4.3	5.5	6.1	78.6		4.5	3.2	6.2	6.2	80.0		3.2	2.3	5.1	6.5	82.9		3.2	3.0	5.3	7.8	80.7	
** 45–59**	2064(29.0)	4.3	4.7	5.4	6.2	79.4		4.0	4.2	5.6	6.3	79.8		2.8	4.1	4.7	6.3	82.1		3.3	3.8	5.9	7.3	79.6	
** ≥60**	2488(35.0)	4.2	3.3	5.1	6.8	80.6		3.9	3.3	4.5	6.9	81.5		3.1	2.8	3.9	6.4	83.8		3.3	2.7	4.7	6.9	82.4	
**Education level**																									
** ≤primary school**	1349(19.1)	6.7	3.4	5.9	6.2	77.8	<0.001	6.0	3.5	6.0	6.3	78.3	<0.001	4.7	3.2	4.4	5.8	81.9	<0.001	5.0	3.4	6.0	7.1	78.6	<0.001
** Middle school**	2061(29.1)	5.5	5.3	6.2	7.1	76.0		4.6	4.9	5.2	7.9	77.4		3.5	4.0	5.6	7.0	79.9		4.2	4.0	5.3	8.3	78.3	
** High school**	2075(29.3)	4.2	4.0	5.8	6.9	79.1		3.6	3.4	6.5	6.3	80.3		2.4	3.1	4.8	7.0	82.7		2.4	3.4	5.6	8.0	80.6	
** ≥Bachelor**	1588(22.5)	2.8	2.3	2.8	5.0	87.2		2.5	2.1	3.3	4.6	87.5		1.6	2.0	2.7	4.4	89.2		1.8	1.6	3.5	4.5	88.6	
**Marital status**																									
5.74.64.66.478.74.84.05.16.379.83.53.93.56.283.03.93.64.66.681.3Married	5739(80.7)	4.6	3.8	5.5	6.4	79.6	0.155	4.1	3.5	5.4	6.5	80.6	0.583	3.0	3.0	4.8	6.3	83.0	0.058	3.2	3.1	5.2	7.3	81.2	0.368
**Medical insuranceNon-married/others1374(19.3)**																									
** Yes**	6348(91.2)	4.7	3.9	5.2	6.0	80.2	0.002	4.0	3.6	5.3	6.0	81.2	<0.001	2.9	3.1	4.4	6.0	83.6	0.038	3.2	3.1	5.1	7.0	81.6	0.053
** No**	610(8.8)	5.9	4.7	6.1	9.1	74.1		5.8	3.3	6.3	9.9	74.7		4.5	3.6	5.4	7.2	79.3		4.9	3.4	5.6	8.6	77.6	
**Financial situation**																									
** Very poor**	473(6.6)	9.4	5.3	4.7	5.4	75.1	<0.001	9.4	5.4	5.0	4.9	75.4	<0.001	6.5	5.6	4.3	5.2	78.4	<0.001	7.5	6.2	5.1	5.6	75.6	<0.001
** Poor**	756(10.6)	6.1	4.4	6.5	6.9	76.1		5.7	3.6	6.3	7.6	76.8		5.0	3.8	4.8	6.4	80.1		4.5	4.1	5.9	8.5	77.0	
** Fair**	3936(55.0)	5.1	3.8	5.2	6.4	79.5		4.3	3.6	5.5	6.5	80.1		3.0	3.0	5.2	6.0	82.9		3.5	2.9	5.5	7.1	81.0	
** Good**	1437(20.1)	2.4	3.5	5.2	7.2	81.8		2.0	3.3	5.2	6.5	83.0		1.6	2.7	4.1	7.3	84.3		1.5	2.8	4.3	7.7	83.7	
** Very good**	549(7.7)	3.4	4.6	5.9	4.6	81.6		3.0	3.2	4.4	5.0	84.4		2.0	2.5	2.5	5.0	84.4		1.8	3.0	4.2	5.4	85.6	

Table 2 shows the distribution in patients’ sense of responsibility across their past hospitalization experience. All these three variables showed significant differences among the four indicators of patients’ sense of responsibility, though in different directions. Greater trust in health professionals predicted greater sense of responsibility to healthcare providers, while greater familiarity with healthcare professionals and hospitalization frequency predicted less patients’ sense of responsibility to healthcare providers.

**Table 2:** Patients’ past hospitalization experience and their sense of responsibility to healthcare providers.

Past hospitalization experience	n(%)	Responsibility to respect doctors						Responsibility to respect nurses						Responsibility to coordinate with medical professionals						Responsibility to comply with hospital rules					
		Don’t know %	Not at all %	Very little %	A little %	Fair/ much %	*p*	Don’t know %	Not at all %	Very little %	A little %	Fair/ much %	*p*	Don’t know %	Not at all %	Very little %	A little %	Fair/ much %	*p*	Don’t know %	Not at all %	Very little %	A little %	Fair/ much %	*p*
**Trust in medical professionals**																									
** **High	5971(72.5)	9.0	6.6	10.4	9.7	64.2	<0.001	8.7	6.0	10.9	10.3	64.3	<0.001	6.5	5.8	9.7	9.7	68.3	<0.001	7.5	5.7	10.8	11.0	65.0	<0.001
** **Low	2267(27.5)	3.3	3.0	3.5	5.2	85.0		2.6	2.7	3.4	5.0	86.3		1.8	2.2	2.8	4.9	88.3		1.8	2.4	3.1	5.7	87.0	
**Familiarity with medical professionals**																									
** **Yes	3602(52.6)	6.2	3.5	3.8	5.1	81.4	<0.001	5.5	3.4	3.9	5.2	82.0	<0.001	4.1	3.1	3.5									
** **No	3250(47.4)	3.4	4.3	7.0	7.5	77.8		3.0	3.7	6.9	7.5	78.9		2.2	3.2	5.8	7.4	81.4		2.1	3.5	6.5	8.8	79.2	
**Hospitalization within the past 3 years**																									
** **Yes	4974(61.5)	4.9	3.4	3.1	5.2	83.4	<0.001	4.1	2.9	3.6	5.0	84.4	<0.001	3.0	2.5	3.1	4.6	86.8	<0.001	3.2	2.7	3.2	5.3	85.6	<0.001
** **No	3114(38.5)	4.8	4.3	6.8	7.1	77.0		4.3	4.0	6.5	7.4	77.8		3.1	3.6	5.6	7.2	80.5		3.4	3.6	6.4	8.3	78.3	

Table 3 displays bivariate logistic regression analysis of patients’ sense of responsibility to healthcare providers. Overall, the results for the four indicators of patients’ sense of responsibility were consistent. Taking responsibility to respect doctors as an example, the strongest predictor of patients’ sense of responsibility was trust in health professionals (odds ratio (OR): 3.24, 95% confidence interval (CI): 2.84–3.69), followed by education level (OR: 2.28, 95% CI: 1.89–2.76). Notably, we found inverse associations of familiarity with health professionals and hospitalization frequency with patients’ sense of responsibility (OR: 0.79, 95% CI: 0.69–0.89; and OR: 0.68, 95% CI: 0.59–0.77; respectively). In addition, unlike single-factor analysis, the impact of financial status on patients’ sense of responsibility to healthcare providers had no statistical significance.

**Table 3:** Social demographic characteristics and past hospitalization experience associated with patients’ sense of responsibility to healthcare providers on logistic regression analysis.

Factors	Responsibility to respect doctors OR(95%CI)	Responsibility to respect nurses OR(95%CI)	Responsibility to coordinate with medical professionals OR(95%CI)	Responsibility to comply with hospital rules OR(95%CI)
**Social demographic characteristics**				
**Gender**				
** Female**	1.11(0.98–1.26)	1.09(0.96–1.24)	1.14(0.99–1.30)	1.12(0.98–1.27)
** Male**	1.00	1.00	1.00	1.00
**Age(years)**				
** ≥60**	1.33(1.13–1.55) **	1.22(1.04–1.44) *	1.22(1.03–1.45) ***	1.26(1.07–1.49) **
** 45–59**	1.21(1.03–1.42) *	1.11(0.94–1.30)	1.07(0.90–1.27)	1.05(0.89–1.23)
** ≤44**	1.00	1.00	1.00	1.00
**Education level**				
1.23(1.06–1.42) **1.24(1.06–1.46) **1.23(1.06–1.43) **Bachelor and above	2.28(1.89–2.76) ***	2.13(1.76–2.58) ***	2.10(1.71–2.57) ***	2.30(1.88–2.80) ***
** Middle school and belowHigh school 1.22(1.05–1.41)** **	1.00	1.00	1.00	1.00
**Marital status**				
** Non-married or others**	1.04(0.88–1.22)	1.05(0.89–1.24)	1.12(0.94–1.34)	1.08(0.91–1.28)
** Married**	1.00	1.00	1.00	1.00
**Medical insurance**				
** Yes**	1.42(1.16–1.73) **	1.46(1.20–1.79) ***	1.34(1.08–1.67) **	1.30(1.05–1.60) *
** No**	1.00	1.00	1.00	1.00
**Financial situation**				
** Good**	0.94(0.78–1.14)	1.01(0.83–1.23)	0.96(0.78–1.18)	1.09(0.89–1.33)
** Fair**	1.09(0.92–1.28)	1.08(0.92–1.28)	1.09(0.92–1.30)	1.16(0.98–1.37)
** Poor**	1.00	1.00	1.00	1.00
**Past hospitalization experience**				
**Trust in medical professionals**				
** High**	3.24(2.84–3.69) ***	3.56(3.12–4.06) ***	3.62(3.15–4.15) ***	3.61(3.16–4.12) ***
** Low**	1.00	1.00	1.00	1.00
**Familiarity with medical professionals**				
** Yes**	0.79(0.69–0.89) ***	0.81(0.72–0.93) **	0.82(0.71–0.94) **	0.77(0.68–0.88) ***
** No**	1.00	1.00	1.00	1.00
**Hospitalization within the past 3 years**				
** Yes**	0.68(0.59–0.77) ***	0.67(0.58–0.77) ***	0.66(0.57–0.77) ***	0.62(0.54–0.72) ***
** No**	1.00	1.00	1.00	1.00

Note

*P<0.05

** *P*<0.01

*** *P*<0.001.

## Discussion

To our knowledge, this study provides the first national evidence regarding patients’ sense of responsibility to healthcare providers in China. Overall, our findings suggest that about one-fifth of participants perceived little or no responsibility to their healthcare providers, especially a responsibility to respect doctors. Although the proportion of these patients was small, their disruptive impact on the working environment and the emotional state of health professionals cannot be ignored. According to an average of 40–60 outpatients attended per day [26], a doctor in China would encounter 8–12 patients daily who perceive little or no responsibility toward them. Of these 8–12 patients, if even just 1 or 2 have a conflict with the doctor, the doctor’s mental and emotional well-being may be disrupted for the entire day or even the next few days. The adverse impact on the working environment and emotional state of health professionals can be represented by the law of the vital few or the Pareto principle (the 80/20 rule) [27–28], which states that approximately 80% of the effects are owing to about 20% of the causes. Additionally, as mentioned above, patients’ sense of responsibility is one of the manifestations of general consumers’ responsibility in the health sector. At the same time, patients are essentially representatives of social groups; therefore, patient's low level or lack of awareness about their responsibility should draw the attention of the health sector as well as society as a whole.

The surge in rights-based movements has empowered people from all walks of life to stand up for their rights; however, we cannot gain some rights at the expense of losing others. Unduly emphasizing the rights of individuals, which has been much exaggerated in the media, can easily lead to individuals becoming too egocentric and neglecting their responsibilities to others. This may be one reason for patients’ low or lacking sense of responsibility to healthcare providers. In fact, from a legal point of view, individuals can give up their rights but they cannot give up their responsibilities. Of course, individual patients and individual doctors should place greater emphasis on their responsibilities to themselves than on their rights. The rights of individuals should be upheld by all kinds of social organizations and agencies, including government agencies, enterprises, hospitals, and schools. Therefore, responsibility-based movements may be more useful as advocates for individuals or pubic.

Among the four indicators of patients’sense of responsibility, the responsibility to coordinate with health professionals was relatively predominant, which may be because it has the closest and most direct relationship with a patient's rights and direct benefits (of eliminating disease or reducing illness). This reveals that patients are conscious of the interrelationship between rights and responsibilities, however this awareness is superficial that only direct situations can be recognized. The positive correlation between patients’ education level and their sense of responsibility may also explain this. Highly educated patients have better medical knowledge and health self-assessment, so they are able to better understand and cooperate with doctors [29]. These findings also reveal, to some extent, that patients are aware of the benefits of fulfilling their responsibilities.

Of all the predictors investigated, greater trust in health professionals best predicted patients’ sense of responsibility to healthcare providers, which validated previous findings from an empirical perspective [30–32]. A patient’s sense of responsibility is a reflection to the patient’s own attitude (as a healthcare consumer) and is also a response to the behavior of his counterparts (as a healthcare provider). Providers of health services cover not only health provider individuals (mainly health professionals) but also health provider organizations; however, health professionals are the primary and immediate objects of patients' sense of responsibility to healthcare providers, which would explain why trust in medical professionals best predicted patients’ sense of responsibility to healthcare providers. This result not only reveals that health professionals should focus on their own ideological and moral accomplishments and behaviors, but also that healthcare provider organizations should adopt more health professional-centered policies.

Subjectively, we may believe that greater familiarity with healthcare professionals and greater hospitalization frequency indicate greater understanding, and therefore, greater sense of responsibility on the part of the patient. However, the findings of empirical analysis in this study were contrary to our prior assumptions. The inverse association between familiarity with healthcare professionals and patients’ sense of responsibility has two plausible explanations. One is owing to the patient's intention, namely, to see a doctor. The patient's focus is on their own illness and related issues; therefore, being acquainted with a health professional helps to reduce the patient’s waiting time for registration and treatment, helps with prioritization for hospitalization, and so on, more so than it helps with gaining a better understanding of the health professional’s job. A second explanation for the observed association is role confusion among patients [33–34]. If patients have no relatives or acquaintances working in the hospital, their role is simply as a patient; conversely, if they do know people working in the hospital, the patients’ role is not limited to being a patient. For example, if a patient’s child is a healthcare provider, the primary role consciousness of the patient would be as a parent rather than as a patient. Further, the role of parents is long-term (formed in the past, and continuing after the patient leaves the hospital); conversely, the role of patients is temporary (newly formed, and automatically dissolved when the patient leaves the hospital) [35–37]. Therefore, within a multi-role environment, the role of patient may be forgotten, and correspondingly, the patient’s sense of responsibility to the healthcare provider would also decrease.

The inverse association between hospitalization frequency and patients’ sense of responsibility has three plausible explanations. One reason is patients’ physical condition and psychological distress. Patients with greater frequency of hospitalization usually have chronic or refractory diseases [36]. They usually begin with high expectations that hospitalization will improve their health status, but these can turn to disappointment when they find that their illness is not cured, and they need to be hospitalized again. Such long-term poor physical condition and psychological distress could arguably lead to a decrease in their feelings of responsibility toward health providers. Another explanation may be related to the almost entirely free choice for patients with respect to seeing a doctor and being hospitalized in China [37], which is like having free choice in a shopping mall. Such a disorderly medical treatment model would make it difficult to establish long-term, stable relationships between patients and healthcare providers [38]. Therefore, more hospitalizations do not promote mutual understanding; on the contrary, such superficial relationships may lead to mutually experienced low levels of trust and responsibility. The impact of trust mentioned above could support this, to a certain extent. The third explanation for the observed inverse association is the patient's excessive and unreasonable expectations of medicine. Patients believe that because they have spent money at the hospital, the doctor should cure their disease, in the same way as spending money in a shopping mall means that a person gets what they want. If the patient's illness is not cured and they need to be hospitalized repeatedly, the patient may attribute the blame to the poor technical skills or the irresponsibility of hospital staff rather than the complexity of their disease.

The positive association between age and patients’ sense of responsibility has two plausible explanations. One is that older patients may feel a greater overall responsibility toward health professionals than do younger individuals [39–40]. The maturity that comes with age and social experience may increase tolerance, kindness, and feelings of responsibility. A second explanation for this association might be that enthusiasm for rights-based movements (as a new phenomenon) is lower among older people [41–42]. Patients without medical insurance showed lower responsibility, which is probably related to the materialistic nature of the patient–provider relationship. These patients may treat the patient–provider relationship as a buyer–seller relationship [43–44]; correspondingly, they may consider that the extent of their responsibility to the provider is to pay the fee.

There are a few limitations of this study that must be noted. First, the response rate was only 33.97%. We did not use any incentives or persuasion to improve participation, as this would increase potential information bias. However, patients’ demographic characteristics in our study were similar to those of other studies with a high response rate in China [45–47]. Second, our survey was cross-sectional, so no conclusions can be drawn about causation. The observed relationships between predictors and patients’ sense of responsibility to healthcare providers should be interpreted as associations.

## Conclusion

In our nationally representative sample, about one-fifth of participants expressed little or no responsibility to healthcare providers in China. However, the lack of respect and collaboration from these patients can negatively affect patient-provider relationships; and such disruptive impact would follow the Pareto principle (i.e., significant effects come from the vital few). Therefore, to improve patient-provider relationships in China, healthcare administrators need to communicate clearly with the patients and the public about the role of patients and the limitations of medicine in order to instill a sense of patients’ responsibility. Additionally, although our findings require validation in different organizational settings, our results suggest that health professionals should be at the center of the effort to build trust and positive relationships with patients.

## Supporting information

S1 File Survey questions. (DOC)
