# Supplementary material for: Patients’ sense of responsibility to healthcare providers and its predictors: A national cross-sectional survey in China
Source: PLoS One. 2018 Dec 5;13(12):e0207361. doi: 10.1371/journal.pone.0207361 (PMC6281211; doi:10.1371/journal.pone.0207361)
Supplement: S1 File — (DOC) [file pone.0207361.s001.doc]

**问题：**

1. **总体上，您认为病人是否有责任尊重医生？**
2. **不知道 （2）无 （3）很小 （4）较小 （5）较/很大**
3. **总体上，您认为病人是否有责任尊重护士？**
4. **不知道 （2）无 （3）很小 （4）较小 （5）较/很大**
5. **您认为病人是否有责任配合医护人员的工作？**
6. **不知道 （2）无 （3）很小 （4）较小 （5）较/很大**
7. **您认为病人是否有责任遵守医院的医疗秩序？**
8. **不知道 （2）无 （3）很小 （4）较小 （5）较/很大**
9. **在您的家人/亲戚中，从事医护工作的有几个？**

**（1） 0个 （2）1个 （3）2个 （4）3个 （5）≥4个**

**6. 不算这次住院，您最近三年总共住院几次？**

**（1） 0次 （2）1次 （3）2次 （4）3次 （5）≥4次**

**7. 您认为现阶段值得信任的医护人员是？**

**（1）很少 （2）较少 （3）一般 （4）较多 （5）很多**

**8. 性别**

**（1）男 （2）女**

**9. 年龄**

**（1）30岁以下 （2）30-44岁 （3）45-59岁 （4）60岁及以上**

**10. 文化程度**

**（1）小学或以下 （2）初中 （3）中专/高中 （4）大学及以上**

**11. 婚姻状况**

**（1）未婚 （2）已婚 （3）离异 （4）丧偶 （5）其他**

**12. 是否有医保**

**（1）有 （2）无**

**13. 您认为自己目前的经济状况如何？**

**（1）很差 （2）较差 （3）一般 （4）较好 （5）很好**

**Survey Questions**

1.“In general, do you think patients have a responsibility to respect doctors?”

(1)I don’t know (2) Not at all (3)Very little (4)A little (5)A fair amount/A lot

2. “In general, do you think patients have a responsibility to respect nurses?”

(1)I don’t know (2) Not at all (3)Very little (4)A little (5)A fair amount/A lot

3. “Do you think patients have a responsibility to coordinate with health professionals in the medical treatment process?”

(1)I don’t know (2) Not at all (3)Very little (4)A little (5)A fair amount/A lot

4. “Do you think patients have a responsibility to comply with hospital rules?”

(1)I don’t know (2) Not at all (3)Very little (4)A little (5)A fair amount/A lot

5. How many of your relatives are health professionals?”

(1)0 (2) 1 (3)2 (4)3 (5)≥4

6. How many times have you been hospitalized in the last 3 years, not including this time?

(1)0 (2) 1 (3)2 (4)3 (5)≥4

7. How many health professionals are trustworthy, in your opinion?

(1)Very few (2) A few (3)Generally (4)Quite a few (5)Many

8. Your gender?

(1)Very few (2) A few

9. Your age(years)?

(1)30 or below (2) 30-44 (3)45-59 (4)60 or above

10. Your education level?

(1) Primary school or below (2) Middle school (3)High school (4) Bachelor’s degree and above

11. Your marital status?

(1) Married (2) Unmarried (3) Divorced (4)Windowed (5)Others

12. Do you have medical insurance?

(1)Yes (2) No

13. What do you think of your financial status?

(1)Very poor (2)Poor (3)Fair (4)Good (5)Very good
